# Blood parasites in vectors reveal a united blackfly community in the upper canopy

**DOI:** 10.1186/s13071-020-04177-0

**Published:** 2020-06-15

**Authors:** Nayden Chakarov, Helge Kampen, Anja Wiegmann, Doreen Werner, Staffan Bensch

**Affiliations:** 1 Department of Animal Behaviour, Bielefeld University, Bielefeld, Germany; 2 Molecular Ecology and Evolution Lab, Department of Biology, Lund University, Lund, Sweden; 3 Friedrich-Loeffler-Institut, Federal Research Institute for Animal Health, Greifswald, Insel Riems Germany; 4 Research Area 2 ‘Land Use and Governance’, Leibniz Centre for Agricultural Landscape Research, Muencheberg, Germany

**Keywords:** Ornithophilic Simuliidae, *Leucocytozoon*, Host-specificity, Vector-driven speciation, Habitat choice, Canopy, Avian malaria

## Abstract

**Background:**

The behaviour of blood-sucking arthropods is a crucial determinant of blood protozoan distribution and hence of host-parasite coevolution, but it is very challenging to study in the wild. The molecular identification of parasite lineages in vectors can be a useful key to understand the behaviour and transmission patterns realised by these vectors.

**Methods:**

In this study, we collected blackflies around nests of three raptor species in the upper forest canopy in central Europe and examined the presence of vertebrate DNA and haemosporidian parasites in them. We molecularly analysed 156 blackfly individuals, their vertebrate blood meals, and the haemosporidian parasite lineages they carried.

**Results:**

We identified nine species of *Simulium* blackflies, largely belonging to the subgenera *Nevermannia* and *Eusimulium*. Only 1% of the collected specimens was visibly engorged, and only 4% contained remains of host DNA. However, in 29% of the blackflies *Leucocytozoon* lineages were identified, which is evidence of a previous blood meal on an avian host. Based on the known vertebrate hosts of the recorded *Leucocytozoon* lineages, we can infer that large and/or abundant birds, such as thrushes, crows, pigeons, birds of prey, owls and tits are the main targets of ornithophilic blackflies in the canopy. Blackfly species contained similar proportions of host group-specific parasite lineages and thus do not appear to be associated with particular host groups.

**Conclusions:**

The *Leucocytozoon* clade infecting thrushes, crows, and pigeons present in most represented blackfly species suggests a lack of association between hosts and blackflies, which can increase the probability of host switches of blood parasites. However, the composition of the simuliid species differed between nests of common buzzards, goshawks and red kites. This segregation can be explained by coinciding habitat preferences between host and vector, and may lead to the fast speciation of *Leucocytozoon* parasites. Thus, subtle ecological preferences and lack of host preference of vectors in the canopy may enable both parasite diversification and host switches, and enforce a habitat-dependent evolution of avian malaria parasites and related haemosporidia.
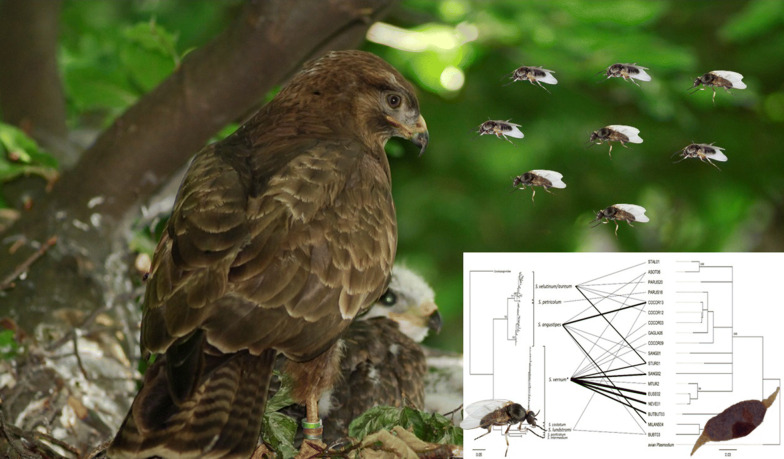

## Introduction

Vector-transmitted parasites are extremely common but insufficiently understood [1]. In theory vectors can both catalyse and hamper the coevolution of hosts and symbionts but observations on the matter are scant [2]. Details of the vector behaviour are crucial for either process but also notoriously difficult to uncover.

Blood-feeding arthropods belong to the major ectoparasitic threats for wild vertebrates, not least because they transmit many potentially life-threatening disease agents [3]. Blackflies (Simuliidae), in particular, can be a major nuisance both due to mass outbreaks and the transmission of pathogens such as trypanosomes, haemosporidians and filarial nematodes [4]. Despite their key role in different environments, research on blackflies has for a long time focused on the accessible aquatic stages [5]. This has resulted in a knowledge gap on the feeding preferences of most species, only slowly improving in recent decades ([6], but see [7]). A substantial challenge to the exploration of the host specificity of most adult blackflies is their strong dependence on natural foraging conditions, which precludes most host choice experiments [4, 8]. However, the development of molecular techniques allows the simultaneous identification of blackflies, the vertebrate blood parasites they contain and the species origin of the engorged blood. Nonetheless, for most simuliids, even of the most common and species-rich subgenera, feeding preferences are known only roughly. Most blackfly groups are so far classified only into mammalophilic or ornithophilic, which is mainly based on basic morphological features and the vertical distribution in the respective habitat [4, 9].

The vertical distribution of blackflies has generally been studied through capture by traps suspended at heights up to 10 m above ground and baited with CO_2_ or live galliform birds (presenting untypical hosts at such heights) [10, 11]. However, in many forests, 10 m is under or the lowermost level of tree canopy, with potentially more than 20 m of canopy above remaining completely unexplored. In the present study, we aimed to identify the blackfly species attracted to birds of prey in their natural habitat, i.e. the upper canopy layers of a central European deciduous forest, as well as their blood diet and the parasitic lineages they carry. We therefore assumed an unorthodox approach and used free-living but stationary raptor broods as a natural bait with a long attraction period.

Simuliids are the principal vectors of avian haemosporidian parasites of the genus *Leucocytozoon*, which was recently shown to have the highest co-speciation rates and highest host-switching rates among blood parasites [12]. These characteristics of *Leucocytozoon* evolution may be assisted by the behaviour of blackflies [13]. The diversity and specialisation of *Leucocytozoon* lineages allow them to be used as natural markers for the diet of the respective vectors [14]. Identifying the genetic lineages of *Leucocytozoon* in blackflies can reveal the diet preferences and behaviour of separate vector species, which in turn can contribute to the understanding of *Leucocytozoon* transmission and evolution [6, 15].

Species of *Leucocytozoon* have been shown to be host-specialised to a certain degree [13]. Within raptors, the sympatric genera of *Accipiter* (hawks) and *Buteo* (buzzards) are hosts of closely related but genetically distinct cryptic species of *Leucocytozoon* [16, 17]. This poses the question if the speciation of *Leucocytozoon* could have occurred in the same habitat due to different simuliid vectors feeding on the different raptor genera [7]. We therefore aimed to sample blackflies around broods of three of the most common birds of prey in central Europe, i.e. common buzzards *Buteo buteo*, northern goshawks *Accipiter gentilis*, and red kites *Milvus milvus*. We predicted that different *Simulium* species will be found around the nests of the three host species, corresponding to the transmitted parasites *L. buteonis* of buzzards and red kites, and *L. mathisi* of goshawks, thus providing an explanation for the high co-speciation rates of *Leucocytozoon*.

On the other hand, almost all species of *Leucocytozoon* have been shown to successfully develop in several tested species of blackflies [15]. Therefore, a potentially unselective prey choice by blackflies may occasionally lead to transmission of *Leucocytozoon* lineages to untypical hosts and facilitate host-switching. Under this scenario, we predicted that *Leucocytozoon* lineages transmitted by blackflies in the same habitat may have distantly related avian hosts.

## Methods

### Study site and sample collection

The study was performed between 2015 and 2017 in eastern Westphalia, Germany (52.05°–52.20°N and 8.30°–8.60°E). The 300 km^2^ study area consists of a hilly landscape dominated by beech forest *Fagus sylvatica*, arable land, with smaller proportions of mixed and coniferous forest (*Picea* sp., *Pinus sylvestris*, *Larix decidua*) and meadows [18]. Forest patches are 0.001–7 km^2^ with a median size of 0.02 km^2^. Small streams intersect nearly every forest patch although many desiccate by July. Few permanent mid-sized streams also flow through the study area. Each study year, all forest patches in the area were searched for active nests of buzzards, red kites and goshawks. Such nests were regularly inspected until nestlings were ca. 3 weeks-old. Between 20th of May and 20th of June, the trees of active nests were climbed, and nestlings were brought to the ground for ringing [19, 20]. During the time when chicks were processed on the ground (20–30 min), insects flying around the focal nest (10–30 m above ground) were caught with a scoop net. Blackflies were stored individually in 100% ethanol.

### DNA extraction, amplification and sequencing

DNA of single female blackflies was extracted from complete specimens using a standard phenol-chloroform protocol and quantified using a NanoDrop spectrophotometer (Thermo Fisher Scientific, Waltham, MA, USA). All samples were screened with three separate PCR assays: (i) blackfly species were determined with conserved primers targeting the cytochrome *c* oxidase subunit 1 (*cox*1) DNA region (LCO1490: 5′-GGT CAA CAA ATC ATA AAG ATA TTG G-3′ and HCO2198: 5′-TAA ACT TCA GGG TGA CCA AAA AAT CA-3′) [21]; (ii) presence of vertebrate host DNA was tested with conserved primers targeting the vertebrate cytochrome *b* (L14841: 5′-AAA AAG CTT CCA TCC AAC ATC TCA GCA TGA TGA AA-3′ and H15149: 5′-AAA CTG CAG CCC CTC AGA ATG ATA TTT GTC CTC A-3′) [22]; and (iii) presence of haemosporidian lineages within the blackfly individual was established with a nested PCR following the protocol of Perez-Martinez et al. [23], using the primer pair Plas1 (5′-GAG AAT TAT GGA GTG GAT GGT G-3′) and HaemNR3 (5′-ATA GAA AGA TAA GAA ATA CCA TTC-3′) for the first PCR and the internal primers 3760F (5′-GAG TGG ATG GTG TTT TAG AT-3′) and HaemJR4 (5′-GAA ATA CCA TTC TGG AAC AAT ATG-3′) for the second PCR. This nested PCR primer protocol amplifies the cytochrome *b* gene of all haemosporidian genera, including raptor *Leucocytozoon* lineages which are less well detected with other nested PCR protocols [24]. Temperature profiles for the PCR reactions were according to [21–23]. PCR products were run on 2% agarose gels. Amplicons were purified with ExoSAP (Thermo Fisher Scientific) and bi-directionally sequenced on an ABI 3730 Analyzer (Applied Biosystems, Waltham, MA, USA) with the BigDye Terminator v1.1 cycle sequencing kit (Thermo Fisher Scientific) using the respective two primers. Raw sequences were edited and aligned in Geneious 8.1.9 (www.geneious.com) and compared with sequences on GenBank, or in the case of 3760F/HaemJR4 with sequences on the MalAvi database, as of 28 January 2020 [24]. Phylogenetic Bayesian inference trees of blackfly and *Leucocytozoon* lineages were created with MrBayes with GTR+G model run for 100 000 generations burnin and 2 million generations post-burnin and subsampled every 500 generations [25].

## Results

We caught 156 blackflies from 64 raptor nests, with 1–14 blackfly individuals caught per nest. Of these 64 nests, 52 were common buzzard nests, 8 belonged to red kites and 4 to goshawks. Only 2 of the blackfly individuals were visibly engorged.

The sequencing of the *cox*1 fragment of blackflies revealed 9 species: 86 individuals belonged to the *Simulium* (*Nevermannia*) *vernum-*group, including the species *S.* (*N.*) *vernum*, *S.* (*N.*) *naturale*, and *S.* (*N.*) *cryophilum*, which are indistinguishable based on the sequenced *cox*1 fragment and will be further referred to as *S.* (*N.*) vernum*. All specimens were caught under 300 masl, which excludes the genetically very similar *S.* (*N.*) *crenobium*, appearing generally above 475 masl [26]. Ten blackfly individuals belonged to *S.* (*N.*) *lundstromi*. A further 35 blackflies were identified as *S.* (*Eusimulium*) *angustipes* and 18 as *S.* (*E.*) *rubzovianum* (formerly known as *S.* (*E.*) *velutinum* [27]). Furthermore, single individuals of the species *S.* (*E.*) *aureum*, *S.* (*E.*) *petricolum*, *S.* (*N.*) *costatum, S.* (*Simulium*) *intermedium* and *S.* (*S.*) *posticatum* were caught around the raptor nests. All specified blackflies had more than 98.5% sequence identity with reference sequences deposited on GenBank. Two specimens could not be genotyped.

The genotyping of vertebrate DNA retained in the blackflies, whether visibly engorged or not, revealed 6 blackfly individuals with vertebrate DNA, of which only 2 were visibly engorged. Three blood meal sources corresponded to common buzzard *Buteo buteo*: one *S. lundstromi* (carrying BUBT03, a *Leucocytozoon*-lineage typical of buzzards); one S. vernum* (carrying ASOT06, typical of owls); and one *S. aureum* (carrying COCOR12, typical of corvids). Another 2 individuals of S. vernum* contained DNA from red kite *Milvus milvus* (not carrying *Leucocytozoon*) and wood pigeon *Columba palumbus* (carrying EUSE02, a *Leucocytozoon*-lineage most closely related to parasites of thrushes), respectively. Furthermore, the only caught *S. intermedium* had fed on *Bos taurus* cattle, corresponding to its known mammalophilic diet [26, 28].

While the traces of blood and corresponding vertebrate DNA in genotyped blackflies provided a limited account of the host spectrum of high-foraging blackflies, the genotyping of parasite lineages delivered a much more comprehensive picture. From the 156 sampled flies, 46 (29.4%) showed amplification of *Leucocytozoon* DNA, inferring that they had previously fed on infected bird hosts. Of these 46 individuals, 25 were identified as S. vernum*, 11 as *S. angustipes*, 5 as *S. rubzovianum*, 3 as *S. lundstromi*, 1 as *S. petricolum*, and 1 as *S. aureum.* The fraction of *Leucocytozoon*-carrying specimens was very similar for all analysed species at ca. 30%.

We were able to identify single infections in 40 of the blackflies, which represented 19 lineages and revealed blackflies to carry parasites typical of thrushes, corvids, birds of prey, owls and tits, in this order of frequency (Table 1).

**Table 1:** *Leucocytozoon* lineages molecularly identified in blackflies of different species, captured close to raptor nests and their respective typical vertebrate hosts

*Leucocytozoon* lineage	Blackfly species						Typical host clade of *Leucocytozoon* lineage^b^
	*S.* (*N.*) vernum*^a^	*S.* (*N.*) *lundstromi*	*S.* (*E.*) *angustipes*	*S.* (*E.*) *rubzovianum*	*S.* (*E.*) *aureum*	*S.* (*E.*) *petricolum*	
MTUR2	1						*Turdus*
NEVE01	4						*Turdus*
STUR1	2			2			*Turdus*
EUSE2	5						*Turdus* (H143, 99%)
SANG1^c^			1				*Turdus* (MTUR01, 95%)
SANG2^c^	3		1				*Turdus* (TUFAL01, 94%)
COCOR03	1						Corvidae, Columbidae
COCOR09	1						Corvidae, Columbidae
COCOR12					1		Corvidae, Columbidae
COCOR13		1	3	1			Corvidae, Columbidae
GAGLA06	1						Corvidae
BUBT03		1	1				*Buteo*
BUTBUT03	3						*Buteo*
MILANS04			2				Accipitridae
ASOT06	1		1				Strigidae
STAL01				1			Strigidae
PARUS18				1			Paridae
PARUS20						1	Paridae
Unidentified/mixed infection	3	1	2				
Total infected	25	3	11	5	1	1	
Not infected	61	7	24	13	0	0	

^a^*S. vernum*, *S. naturale* and *S. cryophilum* are indistinguishable based on the sequenced *cox*1 fragment and are treated together as *S.* (*N*) vernum*

^b^Typical hosts of the respective *Leucocytozoon* lineage are derived from the MalAvi database [24]. Probable hosts of lineages known only from dipteran vectors are derived from BLAST matches. The genetically closest lineage with known vertebrate host and sequence similarity are indicated in parentheses

^c^Lineages described for the first time

**Fig. 1:**
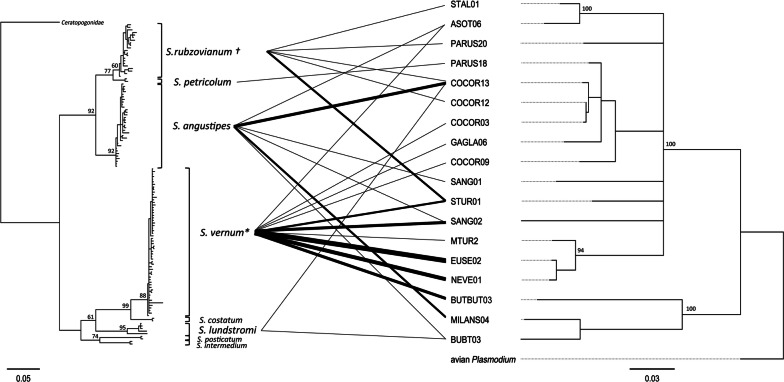
Bayesian inference trees of blackflies, based on 590 bp of the mitochondrial *cox*1 gene, and *Leucocytozoon* lineages carried by these blackflies, based on 504 bp of the mitochondrial *cytb* gene. Blackfly haplotypes are grouped into species (BLAST hits with > 98.5% sequence similarity). Lines connect *Leucocytozoon* lineages and the respective blackfly species in which they were detected. Line thickness is scaled to the number of occurrences. An additional sequence from GenBank was added to species represented by single individuals for better representation. Node support is given for some branches and is based on 1000 bootstrap replicates. † The clade also includes one sequence of *S. aureum*; * *S. vernum*, *S. naturale* and *S. cryophilum* are indistinguishable based on the sequenced *cox*1 fragment and are denoted as S. vernum*

No *Haemoproteus* or *Plasmodium* lineages were detected in the analysed blackflies. Within the represented host groups, there was no obvious association between the identified simuliid taxa with any *Leucocytozoon* lineage or their corresponding host groups (Fig. 1).

The collected blackfly species showed the highest species diversity around nests of common buzzards. *Simulium rubzovianum* was disproportionately more common around nests of red kites, and S. vernum* was more common around nests of goshawks (Table 2, Chi-square test: *χ*^2^ = 45.6, *df* = 6, *P* < 0.001). Nonetheless, no goshawk-specific *Leucocytozoon* lineages could be retrieved from any blackfly individual.

**Table 2:** Number of blackfly individuals of different species captured around the nests of three closely related sympatric raptor host species

Host species nest	Blackfly species			
	*S.* (*N.*) vernum*^a^	*S.* (*N.*) *lundstromi*	*S.* (*E.*) *angustipes*	*S.* (*E.*) *rubzovianum*
*B. buteo* (Common buzzard)	68	10	34	8
*M. milvus* (Red kite)	7		1	10
*A. gentilis* (Northern goshawk)	11			

^a^*S. vernum*, *S. naturale* and *S. cryophilum* are indistinguishable based on the sequenced *cox*1 fragment and are treated together as *S.* (*N*) vernum*

*Note*: Additionally, single individuals of *S. aureum*, *S. costatum*, *S. intermedium*, and *S. posticatum* were caught around nests of common buzzards. One individual of *S. petricolum* was caught around a nest of red kites

## Discussion

Unorthodox approaches can yield unexpected findings about host-vector-parasite interactions. In this case, we aimed to discover whether closely related raptor species attract different simuliid species which may transmit distinct parasite lineages. In the process, we used natural hosts as bait, combined with manual netting. This approach helped to provide new knowledge about the diet of some of the most common European blackfly species. While most studies typically consider only engorged blackflies (but see [7, 14]), we succeeded to derive more information on diets from analyses of the presence of *Leucocytozoon*-lineages in all specimens, combined with existent knowledge on their typical host groups.

### Blackfly species and host preference

One of the main findings of our study was that certain blackfly species forage high up in the canopy and appear to be attracted to avian hosts. All but two out of the 154 identified individuals belonged to the subgenera *Nevermannia* and *Eusimulium*, and nearly 30% of the individuals of both subgenera had fed on birds, as revealed by their *Leucocytozoon* load. This finding indicates that *Nevermannia* and *Eusimulium* are the dominant ornithophilic subgenera of the genus *Simulium* and as such being the most probable vectors of *Leucocytozoon*-lineages in central Europe (c.f. [13]). This corresponds to findings that other European species of *Nevermannia* attack thrushes and warblers, while blackflies of the *S.* (*N.*) *vernum*-group and *S.* (*E.*) *aureum*-group are relatively rare at heights under 10 m in spruce and pine forests [9, 13, 28]. Before this study, feeding preferences of *Nevermannia* and *Eusimulium* species were known only from *S.* (*N.*) *silvestre*, *S.* (*N.*) *curvans*, *S.* (*E.*) *angustipes* and *S.* (*E.*) *aureum*, the latter being one of the best examined ornithophilic blackflies and vectors of *Leucocytozoon* [7, 11, 14, 15, 28, 29]. This species, however, appears to be rather rare in the upper canopy.

The composition of *Leucocytozoon* lineages found in this study shows that *Nevermannia* and *Eusimulium* species attack most avian host groups occurring in this canopy layer, which are sufficiently large and/or abundant, such as thrushes, corvids, pigeons, raptors, owls and tits. A similar size-and-abundance pattern of prey was found among ornithophilic and mammalophilic blackflies close to ground level in Scandinavia [28]. In contrast to the pattern found in Scandinavia, we did not find a strong association between blackfly species or haplotype and *Leucocytozoon* lineages, or their corresponding vertebrate host group [13]. In the study by Hellgren et al. [13], a limited number of engorged *S.* (*N.*) *silvestre* suggested a preference for thrushes. However, a much larger sample of *S.* (*N.*) *silvestre* from North America harboured *Leucocytozoon* lineages infecting avian species across the phylogeny, inferring that this species has a broad range of bird species in its diet [14]. This pattern corresponds much better to our findings from central Europe and supports the notion that species of *Nevermannia* and *Eusimulium* have habitat preferences but are otherwise indiscriminately ornithophilic (Fig. 1, Table 1). Our results deliver no information in which vector species the respective *Leucocytozoon* lineages can complete their development and life-cycle. However, experimental evidence suggests that most parasites of the genus *Leucocytozoon* are more restricted by the ecology of the vector than by its physiology [15].

The absence of non-*Leucocytozoon* parasites also provides insight into the behaviour of blackflies. *Plasmodium* and *Haemoproteus* are not transmitted and cannot fulfil their development in blackflies, but our protocol was apt to detect them, and their prevalence in the putative hosts is relatively high [15, 30]. Therefore, their complete absence confirms that blackflies are not active in the foraging habitat after feeding and remain distant and inactive until the blood meal and potential abortive stages of *Plasmodium* and *Haemoproteus* are digested [4]. Individual blackflies possibly return after oviposition, as the period between two feedings has been measured to take 5–7 days in *S. rugglesi* [31].

### Vector behaviour

To our knowledge, this is the first study of haemosporidian parasites being present in individual non-engorged blackflies. We found that nearly 30% of the blackfly individuals active in the upper canopy layers of central Europe are *Leucocytozoon*-carriers. Blackflies are likely to return to a large stationary food source such as a raptor brood after oviposition [31, 32]. Nonetheless, 30% infected vectors are likely representative of the blackfly population in this habitat, since the raptor lineages potentially belonging to our “bait” accounted for only 15% of all infected blackflies. Previous studies have either analysed pools of non-engorged blackflies or individual visibly engorged blackflies [11, 13, 14, 29]. Engorged blackflies are not active after feeding (0.1–0.3% of all blackfly individuals caught in forests), but can account for up to 23.6% of all blackflies caught with a sweeping net in an alpine habitat [28, 33]. The frequency of *Leucocytozoon*-carriers among freshly engorged blackflies in Scandinavia was 62%, being more representative of the *Leucocytozoon* prevalence in avian hosts, which is expected to be higher there than in central Europe [13, 15]. On the other hand, close to 46% of pools of five non-engorged blackflies seem to contain *Leucocytozoon* lineages, suggesting a *Leucocytozoon* prevalence of approximately 20% in the corresponding blackfly populations [11, 14, 29]. Thus, we complement previous studies of *Leucocytozoon*-carrying blackflies with an individual-based estimate, which may be more precise but is specific to the upper canopy habitat of central Europe.

### Vector and host habitat choice

Finally, we found a substantially different composition of blackfly species around the nests of three closely related avian hosts. *Simulium* (*E.*) *rubzovianum* was overrepresented around nests of red kites, and *S.* (*N.*) vernum* was the only blackfly species present around goshawk nests. At the same time, all species and the greatest diversity were represented around nests of common buzzards. This pattern could be due to host preference. Nests of the three raptor species can potentially be identifiable by odours, which is a primary sense for prey recognition outside of the visible range of blackflies [4, 34]. Buzzards for example, commonly have dead and decaying voles deposited around the nest. Red kites incorporate a great share of carrion and garbage in their nests and food, which lead to a distinct smell of the whole brood. Goshawks, on the other hand, feed mainly on birds and do not keep unconsumed prey remains at the nest. Although blackflies are not attracted to carrion *per se*, these compounds in addition to the native bird odours may enhance distinction of raptor species. However, it seems unlikely that the involved blackfly species discriminate against any of the raptor species, given the patterns outlined by the distribution of *Leucocytozoon* lineages found in this study.

Alternatively, the choice of breeding habitat by the three raptor species may predispose them to a different exposure of blackfly species around their nests. Red kites have a preference for open, dispersed deciduous and mixed forests, while goshawks prefer the core of bigger forests with a higher proportion of coniferous trees, and buzzards cover the whole continuum from single trees to the core of big forests. These preferences may co-vary with the microhabitat foraging preferences of the different blackfly species, which are very poorly known [26]. Such a difference in the blackfly community, however, may have catalysed an ecological speciation of parasites, leading to the cryptic *Leucocytozoon* species infecting currently sympatric raptor species [16, 17, 35]. The substantial difference in sample size between the nests of the three raptor species does not allow us to adequately compare *Leucocytozoon*-lineage diversity around those nests and should be compensated by future studies.

## Conclusions

Vectors are currently the least explored members of host-vector-parasite assemblages [6]. Knowledge of vector ecology and behaviour may be key to understanding the evolution, diversity, prevalence and health impact of parasite populations. Revealing the behaviour of minute arthropods remains challenging and beyond the capacity even of the current bio-logging revolution. Our study approached this aim by relying on the vast knowledge of associations between avian hosts and molecular lineages of blood parasites, which has accumulated in the last decades [24]. This allowed us to discover a distinct community of vectors, which seem to have similar host-seeking behaviour, while being active in the upper forest canopy. It thereby reaffirms the role of parasites as biological markers which can be useful to unveil details of vector biology [15].

## Data Availability

All data supporting the conclusions of this article are included in the article.
